# Structured Ethical Review for Wastewater-Based Testing in Support of Public Health

**DOI:** 10.1021/acs.est.3c04529

**Published:** 2023-08-23

**Authors:** Devin A. Bowes, Amanda Darling, Erin M. Driver, Devrim Kaya, Rasha Maal-Bared, Lisa M. Lee, Kenneth Goodman, Sangeet Adhikari, Srijan Aggarwal, Aaron Bivins, Zuzana Bohrerova, Alasdair Cohen, Claire Duvallet, Rasha A. Elnimeiry, Justin M. Hutchison, Vikram Kapoor, Ishi Keenum, Fangqiong Ling, Deborah Sills, Ananda Tiwari, Peter Vikesland, Ryan Ziels, Cresten Mansfeldt

**Affiliations:** 1 Biodesign Center for Environmental Health Engineering, The Biodesign Institute, Arizona State University, 1001 S. McAllister Ave, Tempe, Arizona 85287, United States; 2 Center on Forced Displacement, Boston University, 111 Cummington Mall, Boston, Massachusetts 02215, United States; 3 Department of Civil and Environmental Engineering, Virginia Tech, 1145 Perry Street, 415 Durham Hall; Blacksburg, Virginia 24061, United States; 4 School of Chemical, Biological, and Environmental Engineering, Oregon State University, 105 26th St, Corvallis, Oregon 97331, United States; 5 School of Public Health, San Diego State University, San Diego and Imperial Valley, California 92182, United States; 6 Quality Assurance and Environment, EPCOR Water Services Inc., EPCOR Tower, 2000−10423 101 Street NW, Edmonton, Alberta T5H 0E7, Canada; 7 Department of Population Health Sciences and Division of Scholarly Integrity and Research Compliance, Virginia Tech, 300 Turner St. NW, Suite 4120 (0497), Blacksburg, Virginia 24061, United States; 8 Institute for Bioethics and Health Policy, Miller School of Medicine, University of Miami, Miami, Florida 33101, United States; 9 Department of Civil, Geological, and Environmental Engineering, University of Alaska Fairbanks, 1764 Tanana Loop, Fairbanks, Alaska 99775, United States; 10 Department of Civil & Environmental Engineering, Louisiana State University, 3255 Patrick F. Taylor Hall, Baton Rouge, Louisiana 70803, United States; 11 The Ohio State University, Department of Civil, Environmental and Geodetic Engineering, 2070 Neil Avenue, 470 Hitchcock Hall, Columbus, Ohio 43210, United States; 12 Department of Population Health Sciences, Virginia Tech, 205 Duck Pond Drive, Blacksburg, Virginia 24061, United States; 13 Biobot Analytics, Inc., 501 Massachusetts Avenue; Cambridge, Massachusetts 02139, United States; 14 Public Health Outbreak Coordination, Informatics, Surveillance (PHOCIS) Office—Surveillance Section, Division of Disease Control and Health Statistics, Washington State Department of Health, 111 Israel Rd SE, Tumwater, Washington 98501, United States; 15 Department of Civil, Environmental, and Architectural Engineering, University of Kansas, 1530 W 15th St, Lawrence, Kansas 66045, United States; 16 School of Civil & Environmental Engineering, and Construction Management, University of Texas at San Antonio, 1 UTSA Circle, San Antonio, Texas 78249, United States; 17 Complex Microbial Systems Group, National Institute of Standards and Technology, 100 Bureau Dr, Gaithersburg, Maryland 20899, United States; 18 Department of Energy, Environmental and Chemical Engineering, Washington University in St. Louis, One Brookings Drive, St. Louis, Missouri 63130, United States; 19 Department of Civil and Environmental Engineering, Bucknell University, Lewisburg, Pennsylvania 17837, United States; 20 Department of Food Hygiene and Environmental Health, Faculty of Veterinary Medicine, University of Helsinki, Agnes Sjöberginkatu 2, P.O. Box 66, FI 00014 Helsinki, Finland; 21 Expert Microbiology Unit, Finnish Institute for Health and Welfare, FI 70600 Kuopio, Finland; 22 Department of Civil Engineering, The University of British Columbia, 6250 Applied Science Ln #2002, Vancouver, BC V6T 1Z4, Canada; 23 Department of Civil, Environmental, and Architectural Engineering, University of Colorado Boulder, UCB 428, Boulder, Colorado 80309, United States; 24 Environmental Engineering Program, University of Colorado Boulder, UCB 607, Boulder, Colorado 80309, United States

**Keywords:** Wastewater-based epidemiology, building-scale, sub-sewershed, ethics, structured review, SARS-CoV-2

## Abstract

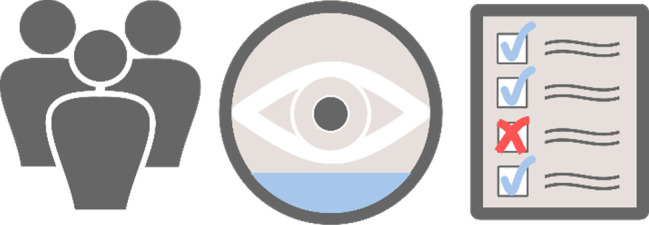

Wastewater-based testing (WBT) for SARS-CoV-2 has rapidly expanded over the past three years due to its ability to provide a comprehensive measurement of disease prevalence independent of clinical testing. The development and simultaneous application of WBT measured biomarkers for research activities and for the pursuit of public health goals, both areas with well-established ethical frameworks. Currently, WBT practitioners do not employ a standardized ethical review process, introducing the potential for adverse outcomes for WBT professionals and community members. To address this deficiency, an interdisciplinary workshop developed a framework for a structured ethical review of WBT. The workshop employed a consensus approach to create this framework as a set of 11 questions derived from primarily public health guidance. This study retrospectively applied these questions to SARS-CoV-2 monitoring programs covering the emergent phase of the pandemic (3/2020–2/2022 (*n* = 53)). Of note, 43% of answers highlight a lack of reported information to assess. Therefore, a systematic framework would at a minimum structure the communication of ethical considerations for applications of WBT. Consistent application of an ethical review will also assist in developing a practice of updating approaches and techniques to reflect the concerns held by both those practicing and those being monitored by WBT supported programs.

## Abstract 2

Development of a structured ethical review facilitates retrospective analysis of published studies and drafted scenarios in the context of wastewater-based testing.

## Introduction: The Need for a Structured Ethical Review

Wastewater-based testing (WBT) describes the sampling of wastewater to support initiatives, such as public health, scientific research, law enforcement, and corporate surveillance. This flexibility of WBT originates from the ability to collect wastewater samples in near real-time, at the population level, and for a variety of analytical targets, including pathogens that cause infectious diseases. Discussing the technique of WBT often progresses rapidly to describing an application rather than exploring the concept in application-free terms.^1^ For example, WBT has been used successfully to monitor enteric and respiratory pathogens, such as poliovirus and coronaviruses.^2,3^ Integrating WBT into the public health surveillance of these pathogens is potentially less invasive, more efficient, and more inclusive than clinical testing. Inclusivity is an inherent property of the aggregated nature of wastewater, which contains target infection bioindicators (e.g., viral RNA) excreted by community members into a municipal sewer network and can capture symptomatic, asymptomatic, and presymptomatic carriers of infectious pathogens regardless of an individual’s access to healthcare.^4^ WBT can also provide an early warning of pathogen presence within a given community as well as detect and track circulating and novel genomic variants (e.g., for SARS-CoV-2).^5−7^ As such, the use of WBT has the potential to be a reliable, cost-effective, objective, and rapid public health tool that complements clinical pathogen testing methods.^8,9^

This initial adoption of WBT, however, challenged well-established public health and research ethical and legal frameworks resulting from the flexibility of the tool. For example, academic researchers who used WBT to monitor pathogens were exempt from, or did not seek, standardized research ethics oversight or review because of the composite nature of the collected wastewater.^10,11^ Whereas public health departments rapidly incorporated wastewater sampling into the surveillance of SARS-CoV-2,^12−14^ WBT additionally supported the research activities of academics and services offered by commercial biotechnology firms. This constellation of state, private, and academic entities is common within the operation and development of public health surveillance, with all entities performing clinical sampling. However, not all sampling translates into surveillance in support of public health, and each entity has separate legal and ethical frameworks.^15^ Therefore, even when considering the ethics of a specific application of the tool, it is important to distinguish between WBT used for public health surveillance, which is measured against well-established professional practice and WHO guidelines,^16^ and with WBT used for nonpublic health purposes (e.g., scientific research, law enforcement) or by nonpublic health entities (e.g., private entities).^17^

When considering WBT in a more application-free context, concerns arise around data utilization and civic governance.^1,15^ Whether WBT is used for research, private monitoring, or public health surveillance, overarching ethical questions have yet to be fully explored. WBT might negatively affect privacy expectations and civil liberties at the community or even individual level.^18−20^ Cases in which wastewater-derived results prompt a targeted response in a specific community could stigmatize or might, in principle, violate the privacy of the sampled community, deflating the unique feature of conducting WBT: anonymity.^21,22^ Conversely, the population at-large has a right to benefit from publicly funded advanced surveillance technologies and the right to information about particular outbreaks so that individuals can make informed decisions about their health,^15^ which ultimately requires broad and transparent communication of WBT results.^23^ Generally, the usage of WBT to monitor for diseases, toxins, and terrorist threats receive broad public support across the United States.^24^ Accordingly, special attention should be paid to contextualize WBT in terms of culture and community values, the intended result of the testing efforts, and the individuals connected to the sewer conveyance network, such that results can be communicated quickly, effectively, equitably, and ethically to maintain community buy-in.^25,26^ A similar point is made by those who advocate for community-based participatory research whenever academic or government investigators seek to learn more about groups of people in a particular or specific region or place. Notably, with the specific sampling place primarily occurring in fixed, publicly owned or operated infrastructure, unique considerations arise in comparison to other public health measures surrounding the civic governance of WBT, necessitating development of a comprehensive framework that captures this multiprofession collaboration.^15^

An ethical framework applied to WBT might limit the challenges that arise in contexts characterized by persistent injustice or violations of human rights. In addition, clearly defined ethical practices and processes can reduce or prevent community harm and resistance to WBT when used for public health surveillance or scientific research purposes. The importance of clear ethical guidelines was recognized by the WHO’s general framework for ethical public health surveillance systems.^16^ Thus, WBT practitioners, which include a mix of scientific experts from diverse disciplines and public health authorities, are increasingly called to translate this general framework into practice,^27^ to both protect the public and ensure a high level of support from the community with broad social acceptance and trust.^28−30^ However, as demonstrated by lack of independent review and oversight of WBT for SARS-CoV-2 monitoring, translating these guidelines into professional practice remains unstandardized and in an early phase of adoption due to those initially conducting WBT having expertise and training outside of public health. Additionally, as WBT continues to expand into further applications that transcend multiple disciplines,^23^ such as opioid detection, monitoring campaigns should be reassessed for each new application, community, and location.^31^ Therefore, interpreting the existing public health frameworks set forth by the WHO and others^15,30,32−34^ and translating them into a concrete, actionable, and specific framework of questions, can assist wastewater practitioners, public health officials, policy makers, utilities, and the public in interpreting the suitability of WBT.

This question-oriented framework can also encourage new entities engaging in the field to rapidly adopt best practices and can provide the tools to identify those applications that fail to align. Critically, the framework is provided as a set of questions to interrogate an application and not a set of finalized guidelines. This framework identifies concerns concretely and rapidly to enable interdisciplinary teams to engage in well-established professional practices in collaboration. Additionally, this framework highlights areas that require further review rather than providing a strict protocol given that ethical issues are often easily raised yet require contextualized analysis and continued engagement by all involved to address successfully. Finally, this standardized set of questions might also promote an ethical research culture, if adopted and upheld as an ongoing practice, and support the reputation and trust of the research field, ensuring equitable and sustainable foundations for WBT systems and community engagement.^35^ For these reasons, we present a structured ethical review framework designed as a worksheet to assist in ensuring successful, long-term, and wide-ranging implementation of WBT.

## Methods: Design of a Structured Ethical Review

Participants contributing to the development of the structured ethical review were recruited through a public announcement at the Water Environment Federation’s Public Health and Water Conference & Wastewater Disease Surveillance Summit on March 23, 2022, as well as active social media announcements and word-of-mouth referrals. From this effort, 29 active participants were involved in the formulation of this study. Participants included representatives from a range of WBT activities, including academic researchers, public health and wastewater practitioners, and private entities working in WBT. The coauthors participating in the framework’s construction represent a broad collection of environmental engineering, public health, and ethics experts that primarily hold or are in training to receive PhDs. Therefore, future application, adaptation, or expansion of this framework would benefit from inclusion of further diverse expertise from other professions, communities, and personal experiences. The workshops drew upon two previously published articles describing ethical considerations of surveillance to develop the framework for a structured ethical review of the existing COVID-19 WBT literature, with the concepts of structured reviews being well-established in the creation of Institutional Review Board (IRB) processes.^36^ The first selected article, written by Gary Marx (1997), emphasizes what the author calls “the new surveillance”.^37^ Marx (1997) poses 29 questions in three categories (the means, the data collection context, and uses) to assess the ethics of surveillance, which the workshop then adapted to better suit WBT. The second article, by Hrudey et al. (2021), explicitly focused on ethical guidelines through 17 questions applied to SARS-CoV-2 wastewater surveillance based on a comprehensive literature review and previous WHO recommendations.^32^ Hrudey et al. concluded that the existing public health ethics literature fails to provide robust guidance for WBT practitioners.

To apply and add to these previous works, the participants developed the structured ethical review framework using a workshop approach. Prior to the workshop, each participant reviewed these two articles on ethics of public health surveillance^32,37^ and drafted concise descriptions for three levels of ethical sufficiency for each of the categories posed by Marx (1997) and Hrudey et al. (2021) applied to WBT activities. The three levels of ethical sufficiency were as follows: 0, minimal review required (no ethical concerns); 1, review suggested (limited ethical concerns); and 2, review strongly suggested (broad ethical concerns). “Review” within the framework indicates that critical further discussion is suggested among stakeholders to explore this category in detail and codevelop best practices. These levels were selected to prioritize ethical reviews and the communication efforts of those designing and operating surveillance programs. Thereafter, each participant independently filled out their brief written descriptions within a shared document for each previously identified category of ethical consideration identified in the two articles. During the virtual workshop, participants reviewed the responses and identified the guidelines and questions that warranted further discussion; the output thus identified consensus descriptions of ethical sufficiency rankings. After the workshop, each participant adopted a category and prepared a final draft description for the three levels of ethical sufficiency into a table (Supporting Table 1). All coauthors then reviewed the table, and revisions were made until a consensus final draft was reached, with duplicate categories merged but all others preserved from Marx (1997) and Hrudey et al. (2021). The final, fully consolidated framework comprises 37 categories of ethical consideration for WBT, with three ordinal ranking descriptions within each category (Supporting Table 1). These categories broadly represent key considerations in community engagement, equality, establishment of a new precedent, and data integrity. With the large size of the framework, the set was further refined, with the participants being asked to list ten essential questions to include. In total, 16 participants provided a score, and those categories receiving more than 9 votes or higher were included in the final framework (Table 1). In essence, the developed structured ethical review provides a set of questions that enables users to provide a score (higher the score the greater the ethical concern) when considering a WBT application.

**Table 1:** Consolidated Framework for a Structured Ethical Review to Assess Potential Adverse Outcomes of WBT Efforts^a^

Category	0—Minimal Review Required	1—Review Suggested	2—Review Strongly Suggested
**Legitimacy:** Are surveillance data collected only for a legitimate public health purpose?^†^	Data support public health agents for public health measures	Data support nonpublic health agents for public health measures	Data support nonpublic health agents (or public health agents acting outside of public health) for nonpublic health purposes
**Unfair Disadvantage:** Is the information used in such a way as to cause unwarranted harm or disadvantage to its subject?*	WBT is implemented with clear scope, oversight, and decision-making procedures including procedures for response to wastewater data with policies for follow-up clinical testing applicable to full communities	Unintentionally subjecting specific areas to the possibility of a disruptive intervention (lockdowns, quarantines, isolations of entire areas without any process for identifying and isolating relevant individuals; mandatory testing of individuals in response to wastewater data) whereas excluding others from the same level of surveillance scrutiny and response	Intentionally subjecting specific areas to the possibility of a disruptive intervention (lockdowns, quarantines, isolations of entire areas without any process for identifying and isolating relevant individuals; mandatory testing of individuals in response to wastewater data) whereas excluding others from the same level of surveillance scrutiny and response
**Data Stewardship and Protection:** Is the data properly maintained to protect those monitored?*	Data managed per requirements of and for community monitored (codeveloped with the community and professional practice)	Data managed per requirements for community monitored (set by professional practice alone)	Data management plan absent
**Creation of Unwanted Precedents:** Is it likely to create precedents that will lead to its application in undesirable ways?*	Analysis for individual identification prohibited; explorations outside of agreed-upon community scope is explicitly prohibited	No positional statement regarding individual identification; No discussion of future research is discussed	Explicitly for identification of individuals or otherwise unethical applications
**Awareness:** Are individuals informed they are being monitored and why?*	Representative(s) of the monitoring campaign are in a cycle of continued community outreach and engagement during WBT and over the clearly defined reporting period providing contextualization of the scope and intent to minimize misrepresentation or misuse; those monitoring capture the questions from the community rather than the wastewater utility operators	Duration, scope, and intent is communicated and disseminated in a passive manner without contextualization or engagement OR communicated to a single representative of the community; the wastewater utility operators respond to increased inquiries but have access to those collecting the data to direct inquiries	No direct communication of the duration, scope, and intent to the monitored community members; the burden of communication falls solely on the third-party wastewater utility operators rather than the data collectors
**External Data Sharing:** Is the public health surveillance data shared with other public health agencies when addressing a public health need?^†^	Collected WBT-supported public health data is shared freely with/between public health agencies when a public health need presents or persists	Collected WBT-supported public health data only partially shared with/between public health agencies when a public health need presents or persists	No data is shared when a public health need presents or persists
**Public Decision-Making:** Was the decision to use WBT in surveillance arrived at through some public discussion and decision-making process?*	Surveillance program was designed in a public manner (e.g., review by elected officials and public through town halls for initial implementation and continued operation) with a good-faith effort to reach both those receptive or resistant to the objectives of public health	Program does not receive formal public authorization but is broadly supported by the public (as informed by representative public surveys)	Program does not receive formal public authorization and is not supported by the public (as informed by representative public surveys)
**Right of Inspection:** Are people aware of the findings of WBT supported surveillance and how they were created?*	Representative(s) of the monitoring campaign are in a cycle of continued community outreach and engagement during the sample collection and reporting period providing contextualization of the collected data to minimize misrepresentation or misuse (e.g., updating an annotated and agreed-upon Internet-accessible dashboard; timely and routine public town-halls or open seminars; direct mailing to surveyed individuals)	The collected data is communicated and disseminated in a passive manner without contextualization or engagement OR communicated to a single representative of the community	No direct communication of the collected data to the monitored community members
**Equality-inequality:** Is WBT broadly applied to all or only those able to resist?*	Entire community is monitored (e.g., treatment plant; jail sampling that monitors the effluent of the whole jail including staff and inmates)	Representative coverage is achieved (e.g., manhole sampling, but ensuring that demographics of surveilled communities are representative of the entire city; jail sampling that has sites for staff and inmates separately)	Only protected-class communities are monitored (e.g., manhole sampling that surveils only low-GDP per capita areas; jail sampling that only surveils inmates)
**Community Values:** Are the values and concerns of the communities taken into account in planning, implementing, and using data from surveillance?^†^	Representative of the monitoring campaign are in a cycle of continued community outreach and engagement during the planning and implementing period to address the concerns and support the values of the community	Representative of the monitoring campaign are engaged during the planning period only to address the concerns and support the values of the community	No direct involvement of the monitored community members
**Consequence of Inaction:** What are the consequences of taking no surveillance action?*	Mortality, morbidity, or other adverse effects is imposed on community by lack of surveillance	The surveillance data does not minimize adverse effects to the community	The community benefits by not being surveilled

a Specific categories originated from either Marx 1997^37^ (*) or Hrudey et al., 2021^32^ [^†^]. “Review” within the framework indicates that further critical discussion is suggested among stakeholders to explore this category in detail and codevelop best practices. The top 11 categories based on the internal voting are presented here, and the full framework is presented as Supporting Table 1, also available at DOI: 10.17632/2xkfkcsxx8.1.

### Inclusion Criteria for Published Studies Considered within the Application of the Structured Ethical Review

The initial collection of studies was obtained by searching for articles containing the keyword phrases “SARS-CoV-2” and “wastewater”, yielding a total of 5,632 articles as of February 2022. To focus on sampling strategies that potentially challenge individuals’ assumptions of privacy, further filtering was performed to include studies reporting on near-building and/or within-sewer monitoring. This involved incorporating additional modifiers, such as “campus”, “nursing home not campus”, “prison not campus”, “hospital and wastewater-based and building not campus”, and “neighborhood not campus”. Consequently, the modified search categories returned 1204, 240, 119, 201, and 313 articles, respectively. Studies were excluded if they did not mention monitoring within a sewer collection system at the neighborhood or building-level scale or if they had not yet been published as peer-reviewed articles. This strategy was used to narrow the database into a representative and manageable subselection of WBT related literature covering the early phase of the pandemic as a case study. The goal here was to emphasize the utility of the ethical framework applied to a coherent set of WBT applications rather than to define the best practices for a given application. Therefore, this structured review approach could be applied in the future to private residential settings^38^ or at wastewater treatment plant scales,^39^ but both areas were excluded from current consideration.

After applying these filters, 25 near-building and 21 within-sewer SARS-CoV-2 WBT studies were identified for review. The inclusion of 7 additional articles published after the initial search that were previously in preprint status increased the number of studies included in this structured ethical review to 53 (56 publications were represented by these studies, with 3 having two publications describing the same monitoring campaign and were considered in concert). While published research articles inherently connote research use of WBT, the selected articles had potential direct applications to public health, facilitating their use in the evaluation of the structured ethical review framework.

Papers were divided across participants for review with overlapping assignments provided. Overall, 79 reviews were completed by answering all 37 questions based on the reviewers’ analysis of the presented text alone. Conflicting assignments were evaluated but were left unchanged, resulting in an average score for those papers. The reviews were then consolidated into a summary data frame for broad comparisons between the responses to individual questions.

## Results and Discussion

### Reconsidering SARS-CoV-2: Application of the Structured Ethical Review

**Figure 1:**
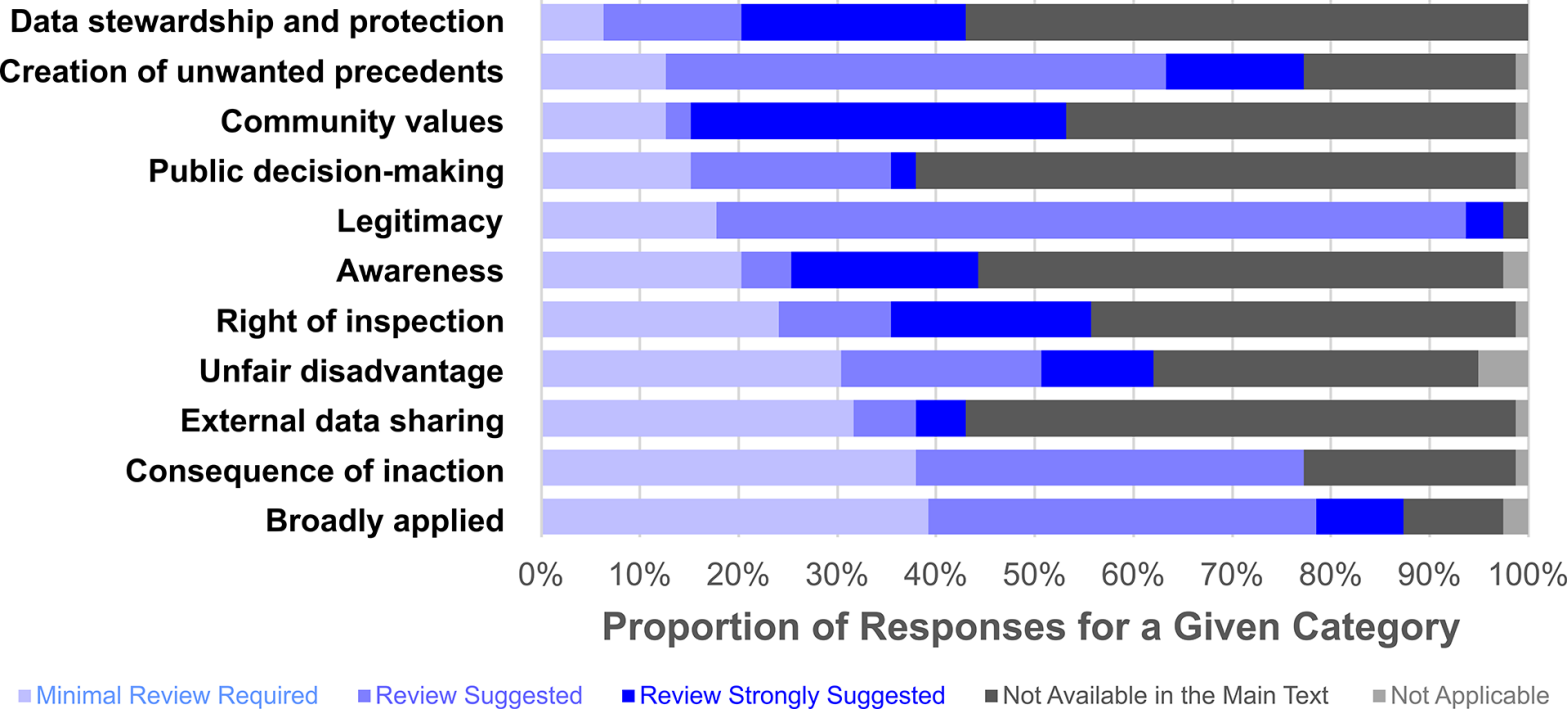
Distribution of assigned flags (“minimal review required”, “review suggested”, “review strongly suggested”, “not available in the main text”, “not applicable”) for the top 11 categories in the structured ethical review, represented as a fraction percent of all publications analyzed (*n* = 53) with multiple reviewers providing reports for select individual studies, resulting in more reviews than studies (*n* = 79).^12,42−46,50−99^ In total, 56 publications were represented by these studies, with 3 having two publications describing the same monitoring campaign and considered in concert. The categories are sorted by ascending proportion of “minimal review required”.

The main goal of this study was to develop a framework to assist in identifying gaps in ethical considerations of the WBT as a tool. This distinction between WBT as a tool throughout the text is deliberate to distinguish it from the more often reported wastewater-based surveillance (WBS). “Surveillance” is a term of art and, in the context of public health, describes a method of learning about health, which is different from research or other applications of WBT. “Testing” is the technology or tool that can be used for a number of processes including research, public health surveillance, or other types of monitoring. Therefore, this study developed and then demonstrated a structured approach to considering WBT applications by applying the final framework to previously reported SARS-CoV-2 WBT studies (Figure 1).

Application of the structured ethical review framework highlighted that throughout the published articles analyzed in this work, gaps in information were observed in both the manuscript and supplemental information (1247 of 2923 answers provided (43%) indicated “Not Recorded in the Main Text”). This absence may be the result of the inclusion of studies predominantly reported by those not directly working for public health departments (e.g., external academics and researchers). For example, when compared to clinical research in which detailed informed consent (i.e., voluntariness, information disclosure, decision-making capacity, and communication of results) is required,^40^ few WBT studies articulated whether consent was obtained from the studied community, whether those results were communicated back to the community, and/or whether those results were used with clear public health objectives and outcomes. It is important to note that the WBT can be applied as a public health surveillance tool that would then operate within existing legal and regulatory frameworks. Within these specific applications, consent for the collection and testing of wastewater samples may not be required from individuals when a pooled sample is being analyzed, as this is considered to be part of routine public health surveillance.^41^ However, there may be legal and ethical considerations around the use of the WBT data collected, for example, in research applications, and these should be addressed by relevant authorities and other frameworks. Broadly, the absence of reported information for evaluating community engagement and data safeguards reflects the current lack of a standardized ethical framework for WBT campaigns.

This exemption from reporting and lack of oversight may also be due to a lack of clarity for the motivation of the WBT study. For example, these articles could have been originally motivated by research interest with results that prompted a public health action. Conversely, authors may have retrospectively published results from a public health surveillance system. In the former case, authors may have assumed the research study was exempted from IRB approval given the composite nature of the sample, which is believed to prevent the ability to identify specific individuals in a given sewer catchment.^42^ In certain cases, authors referenced IRB approvals for utilizing individual case data,^43^ but in most cases, WBT sample data itself was determined to be exempt from IRB oversight. In select cases, ambiguity surrounded whether the IRBs themselves arrived at these determinations or the researchers, a key consideration given that these oversight bodies are responsible for conforming to federal regulations. Within the Code of Federal Regulations, agencies such as the Department of Health and Human Services (Title 45), Food and Drug Administration (Title 21), and Environmental Protection Agency (Title 40), stipulate the composition, function, and requirements for IRBs conducting funded research.

However, the data collected from WBT were sometimes used for direct public health interventions, a usage of WBT that aligns with public health surveillance and is distinct from research activities governed by IRB protocols. For example, other studies (mainly dormitory and hospital surveys) reported on positive SARS-CoV-2 detection in wastewater, triggering mandatory clinical or individualized testing from which infected individuals were identified.^43−46^ In contrast, WBT of a cargo ship specifically explored border protection against infected seafarers as a potential public health intervention,^47^ a more intervention-driven approach than that described as a complementary monitoring tool for airplanes.^48^ Ultimately, the study did not support WBT in the cargo ship application, notably because individualized testing was already deployed. This rapid application of data highlights a key finding: the motivation for the use of WBT during the early phase of the pandemic was necessarily blurred between research and public health surveillance. Needed investigation into WBT was required to demonstrate the utility of the signal to public health practice. Research goals were coupled with the intent of researchers to help communities face the threat of a pandemic, notably on university campuses of and neighboring communities to the researchers conducting the testing.

This dual nature of early monitoring campaigns complicates their interpretation. For example, if WBT was conducted as part of a public health surveillance effort, informing subsequent action where individuals were identified through individualized testing regimes (e.g., isolation), then IRB oversight would be unnecessary, as this use of WBT would fall under ethical guidelines for public health surveillance. This determination that IRB is not required rests on WBT being used as a tool for public health surveillance conducted by a public health authority, which is explicitly excluded from the US IRB regulations (Subpart A of 45 CFR Part 46).^49^ However, the IRB should still review other applications of WBT aimed at producing generalizable knowledge focused primarily on a research purpose rather than public health surveillance (see Categories 4, 10, and 28 in Supporting Table 1). Notably, none of the 53 studies reviewed here, which were published primarily in environmental-focused journals, reported that their institutions required IRB approval for the WBT portion of their research. However, this does not necessarily indicate that IRB was not required or performed. In compliance with IRB regulations, studies that pair WBT with public health surveillance should seek IRB review if research aspects of the work are expected. Furthermore, researchers must provide comprehensive information and potentially contextualization when submitting materials to the IRB to ensure that panel members with a diverse set of expertise can evaluate the merits and concerns of the study effectively.

In contrast to the ambiguity surrounding IRB review reporting, the WBT community has already taken to reporting on other ethical areas including the scale of testing (e.g., wastewater treatment plants [WWTPs], building-level), the identifiability of populations represented, the presence/absence of validated QA/QC workflows, and the need for clear statements of goals supporting public health. However, gaps in presenting ethical considerations were found with respect to stakeholder participation in the development and deployment of WBT efforts, as well as data and sample management. Only 5 of 53 studies clearly identified a data management plan, with no study combining an additional communication or engagement plan. Elements that were considered when screening for a communication or engagement plan included statements surrounding how or whether the public or public representatives were engaged in the development, deployment, or future applications of WBT; the surveilled parties knew their rights or were given the right to challenge, express grievances, or seek redress; and if the potential risks and/or benefits were outlined in detail to these populations or third parties. It is possible that some studies developed communication and engagement plans that were not explicitly reported in published research, and we acknowledge this limitation in our review. Further, wastewater data security, handling, and subsequent use in and outside the scope of the project (including the fate of remaining samples) were largely neither acknowledged nor discussed. Details on data ownership, security, management plans, and dissemination were generally absent, but key elements were provided in select studies.^44,51,82,96,98^ Several elements that require more explicit elaboration include how data were reported during the operation of the campaign, by what mechanism, at what frequency (weekly, biweekly, etc.), and how thresholds of concern were established.

In the case of campus- or dormitory-wide testing at colleges and universities, authors provided more information for these programs when compared to studies for large sewersheds, for instance, at the level of a WWTP. Specifically, these studies detailed follow-up procedures for wastewater samples that resulted in positive detection of the virus (e.g., lockdowns, contact tracing), stakeholder engagement, and data dissemination plans. Likely, this higher level of detail resulted from building-level monitoring programs intentionally designed to use WBT to assist in clinical testing and quarantine procedures. However, of all papers reviewed, only a few noted a process for obtaining consent from the studied populations.^45,50,63,78,80,84,90,99^

### Learning from SARS-CoV-2: Sustaining Future Applications of WBT

The structured ethical review aims to provide a framework to assess new areas or targets of monitoring and to document evolving ethical applications of WBT when uniting technical innovation, community engagement, and professional collaboration.^100^ For instance, the unique characteristics of the most recent orthopoxvirus outbreak, which differs in transmission classification and carries higher pre-existing stigma, present additional ethical considerations within WBT because of its primary circulation in men who have sex with men.^101^ Additional considerations must be brought to bear that weigh private access to medical interventions, broad public scrutiny of communication, and the self-determination of historically marginalized groups. Further future applications will present additional new and unique challenges to the ethical application of WBT as a tool, promoting a continuous evaluation of the structured ethical review adopted here. Therefore, establishing, applying, and updating this structured ethical review over time will result in a transparent record of our understanding of the best ethical practices when applying WBT within and beyond public health surveillance.

Future WBT structured ethical reviews may need to consider human-specific, rather than just pathogen-specific, target biomolecules. Early within the development of this structured ethical review, it was established that the purview of recovering human genetic material was not the main focus of this tool given that considerations for targeted human monitoring are already developed in the biomedical research community and remain a bioethics concern beyond that of WBT.^102^ However, with the technical capabilities of WBT advancing, the application of next-generation sequencing tools to collect personally identifiable health information opens unique and potentially community-desired possibilities.^103^ Although relatively few papers used sequencing techniques and, when applied, were exercised only for detecting variants of SARS-CoV-2 in which human-specific DNA is masked from publicly posted samples, there is an evident lack of guidelines when analyzing complete genetic data recovered from wastewater.^18^ Notably, previous applications successfully applied more targeted approaches to screen for human mitochondrial sequences within wastewater as a population biomarker highlighting outside of sequencing technologies.^104^ This necessitates a better understanding of the views and tolerances of those conducting targeted human-DNA testing, those using the data, and persons whose samples are being tested,^105^ potentially informing the evolution and revision of the categories within the structured ethical review.

Importantly, this framework does not define which applications of WBT are ethically appropriate and which are not; it is simply a tool to guide the development and evolution of WBT campaigns by highlighting aspects that may require additional ethical review. In an ideal world, all WBT campaigns would receive ratings of “minimal review required” for all categories, but in practice, this will almost certainly never be the case. Applying WBT requires trade-offs to maximize the benefit and minimize harms. For example, waiting to implement WBT for a novel pathogen until all ethical concerns are fully resolved may hinder progress in public health surveillance to identify new outbreaks and intervene in their early stages. Different categories within the framework can also be in tension with one another, which is common for large frameworks and thus is expected. Additionally, the interpretation of each category will change accordingly as sampling campaigns are run by or analyzed from the viewpoint of researchers, governmental public health agencies, or private entities.

The role and utility of ethics analysis in public health, research, and clinical practice is rarely to give approvals or disapprovals of inherently challenging issues and conflicts. It is, rather, to inform an already complex environment or problem by making clear salient values–some of which might be in conflict–and help practitioners weigh and apply these values. How, for instance, should scientists navigate a duty to protect privacy with the right for the monitored individual and public to benefit from WBT? The challenge occurs when reasonable people disagree about an appropriate course of action. In such cases, the existence of an objective and transparent ethics process can guide both investigators and communities such that whatever action is decided there will be a mutual understanding of the cause. It is our recommendation that WBT practitioners and the served communities adopt structured ethics reviews to facilitate this process.

As WBT expands and new practitioners and researchers enter the field, this structured ethical review framework will provide education and guidance to promote best practices.^106^ Given the length of the full, structured ethical review in terms of the number of categories, further development surrounding ease-of-use is required to ensure wide-scale adoption of the structured ethical review framework by the WBT community. Furthermore, the framework is designed as a living document, allowing stakeholders to assess, challenge, and revise the framework and understanding of ethical practice for specific applications. This adaptive, iterative approach to improving the framework is critical for managing this technology in the future, safeguarding the well-being of those under surveillance, and communicating robust ethical guidelines to protect the intended applications. Therefore, we strongly recommend the implementation and adaptation of this structured ethical review by all those involved in both ongoing and future WBT campaigns to promote activities supporting ethical best practices.

## Supporting Information Available

Bar chart summarizing the responses for the full Structured Ethical Review framework and table capturing the full Structured Ethical Review framework (DOCX)
