# Supplementary material for: Structured Ethical Review for Wastewater-Based Testing in Support of Public Health
Source: Environ Sci Technol. 2023 Aug 23;57(35):12969–80. doi: 10.1021/acs.est.3c04529 (PMC10484207; doi:10.1021/acs.est.3c04529)
Supplement: Supplementary file 1 — es3c04529_si_001.docx [file es3c04529_si_001.docx]

**Supporting Information for Structured Ethical Review for Wastewater-Based Testing in Support of Public Health**

Devin A. Bowes^1,2*^; Amanda Darling^3*^; Erin M.Driver^1*^; Devrim Kaya^4,5*^; Rasha Maal-Bared^6^; Lisa M. Lee^7^; Kenneth Goodman^8^; Sangeet Adhikari^1^; Srijan Aggarwal^9^; Aaron Bivins^10^; Zuzana Bohrerova^11^; Alasdair Cohen^2,12^; Claire Duvallet^13^; Rasha A. Elnimeiry^14^; Justin M. Hutchison^15^; Vikram Kapoor^16^; Ishi Keenum^17^; Fangqiong Ling^18^; Deborah Sills^19^; Ananda Tiwari^20,21^; Peter Vikesland^3^; Ryan Ziels^22^; Cresten Mansfeldt^23,24†^

*DAB, AD, EMD, and DK contributed equally to this work

^†^Corresponding author: [cresten.mansfeldt@colroado.edu](mailto:cresten.mansfeldt@colroado.edu)

1. Biodesign Center for Environmental Health Engineering, The Biodesign Institute, Arizona State University, 1001 S. McAllister Ave, Tempe, AZ, 85287, USA
2. Center on Forced Displacement, Boston University, 111 Cummington Mall, Boston, MA, 02215, USA
3. Department of Civil and Environmental Engineering, Virginia Tech, 1145 Perry Street; 415 Durham Hall; Blacksburg, VA 24061, USA
4. School of Chemical, Biological, and Environmental Engineering, Oregon State University, 105 26th St, Corvallis, Oregon 97331, USA
5. School of Public Health, San Diego State University, San Diego and Imperial Valley, CA, USA
6. Quality Assurance and Environment, EPCOR Water Services Inc., EPCOR Tower, 2000–10423 101 Street NW, Edmonton, Alberta, CA
7. Department of Population Health Sciences and Division of Scholarly Integrity and Research Compliance, Virginia Tech, 300 Turner St. NW, Suite 4120 (0497), Blacksburg, VA 24061, USA
8. Institute for Bioethics and Health Policy, Miller School of Medicine, University of Miami, Miami, Florida, 33101, USA
9. Department of Civil, Geological, and Environmental Engineering, University of Alaska Fairbanks, 1764 Tanana Loop, Fairbanks, AK 99775, USA
10. Department of Civil & Environmental Engineering, Louisiana State University, 3255 Patrick F. Taylor Hall, Baton Rouge, LA, 70803, USA
11. The Ohio State University, Department of Civil, Environmental and Geodetic Engineering, 2070 Neil Avenue, 470 Hitchcock Hall, Columbus, OH, 43210, USA
12. Department of Population Health Sciences, Virginia Tech, 205 Duck Pond Drive, Blacksburg, VA 24061
13. Biobot Analytics, Inc., 501 Massachusetts Avenue; Cambridge, MA, 02139, USA
14. Public Health Outbreak Coordination, Informatics, Surveillance (PHOCIS) Office – Surveillance Section, Division of Disease Control and Health Statistics, Washington State Department of Health, 111 Israel Rd SE, Tumwater, WA, 98501, USA
15. Department of Civil, Environmental, and Architectural Engineering, University of Kansas, 1530 W 15th St, Lawrence, KS, 66045, USA
16. School of Civil & Environmental Engineering, and Construction Management, University of Texas at San Antonio, 1 UTSA Circle, San Antonio, TX, 78249, USA
17. Complex Microbial Systems Group, National Institute of Standards and Technology, 100 Bureau Dr, Gaithersburg, MD, 20899, USA
18. Department of Energy, Environmental and Chemical Engineering, Washington University in St. Louis, One Brookings Drive, St. Louis, MO, 63130, USA
19. Department of Civil and Environmental Engineering, Bucknell University, Lewisburg, PA, 17837, USA
20. Department of Food Hygiene and Environmental Health, Faculty of Veterinary Medicine, University of Helsinki, Agnes Sjöberginkatu 2 P.O. Box 66 FI 00014 Helsinki, Finland
21. Expert Microbiology Unit, Finnish Institute for Health and Welfare, Kuopio, Finland
22. Department of Civil Engineering, the University of British Columbia, 6250 Applied Science Ln #2002, Vancouver, BC V6T 1Z4, CA
23. Department of Civil, Environmental, and Architectural Engineering, University of Colorado Boulder, UCB 428, Boulder, CO, 80309, USA
24. Environmental Engineering Program, University of Colorado Boulder, UCB 607, Boulder, CO, 80309, USA

**Summary: 8 pages, 1 Figure, 1 Table**


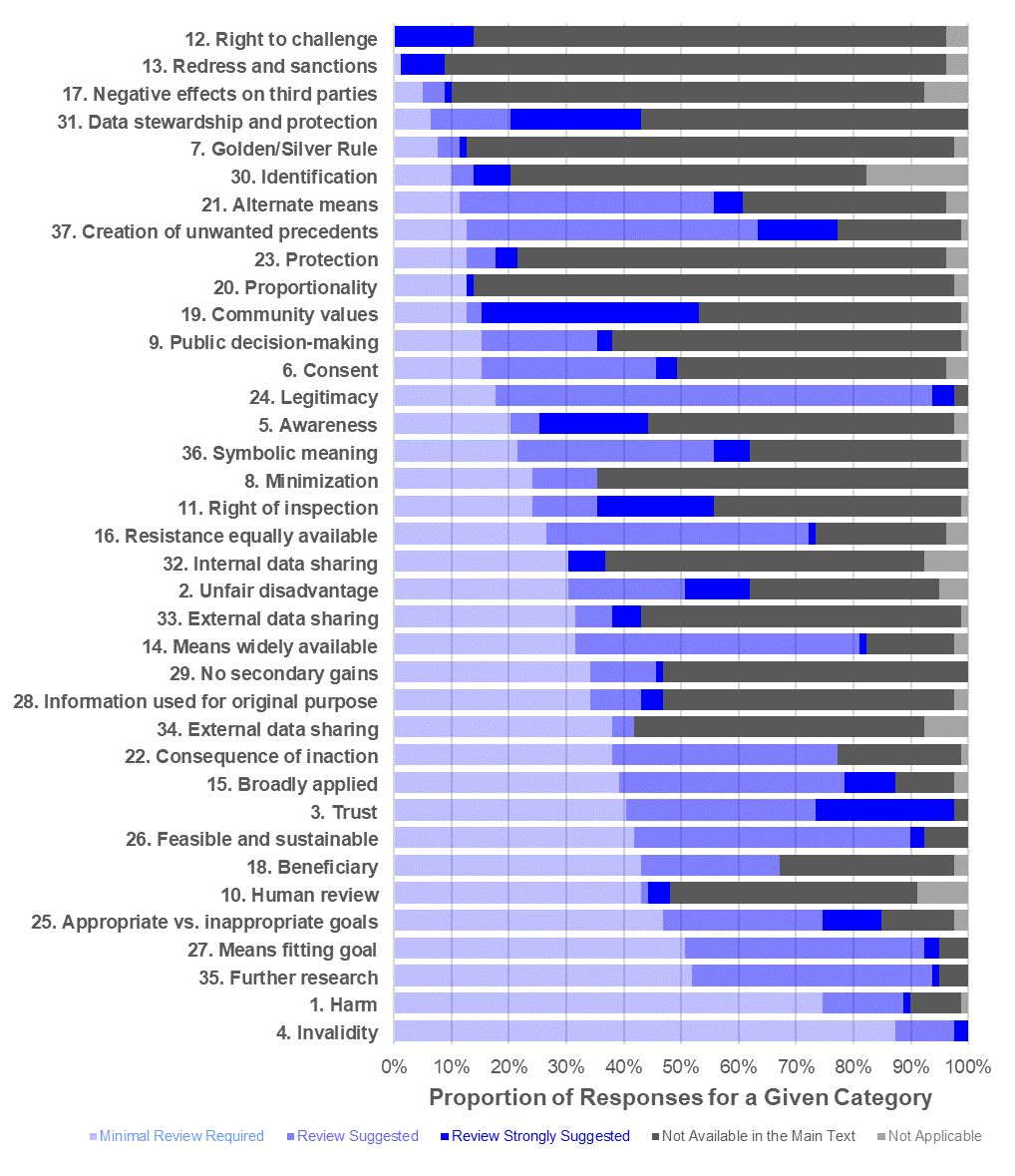


**Supporting Figure 1.** The distribution of assigned flags (“minimal review required”, “review suggested”, “review strongly suggested”, “not available in the main text”, “not applicable”) for each structured ethical review category represented as a fraction percent of all publications analyzed (n=53) with multiple reviewers providing reports for select individual studies, resulting in more reviews than studies (n=79) ^1,33–37,40–89^. The categories are sorted by ascending proportion of “minimal review required”.

**Supporting Table 1.** Full framework for a structured ethical review to assess potential adverse outcomes of WBT efforts. Specific categories originated from either Marx 1997 [*] or Hrudey et al., 2021 [†]. ’Review’ within the framework indicates that further critical discussion is suggested among stakeholders to explore this category in detail and codevelop best practices.

| **ID** | **Category** | **0 - Minimal Review Required** | **1 - Review Suggested** | **2 - Review Strongly Suggested** |
| --- | --- | --- | --- | --- |
| 1 | **Harm:** Does WBS cause unwanted physical or psychological harm?* | 0 - Data are anonymized/inherently anonymous to prevent harm to individuals; a risk analysis is performed to explore potential harm to communities in the form of discrimination or other unintended intervention and finding none; proper safety adherence for sample collection | 1 - Data are not anonymized and may be used against individuals and communities for surveillance targets without previously established associated stigmas; safety training of sample collection ambiguous | 2 - Data are not anonymized and may be used against individuals and communities for surveillance targets with known associated stigmas; safety training of sample collection absent OR noted excess risk of harm to wastewater professionals |
| 2 | **Unfair Disadvantage:** Is the information used in such a way as to cause unwarranted harm or disadvantage to its subject?* | 0 - Programs implemented with clear scope, oversight, and decision making procedures including procedures for response to wastewater data with policies for follow up clinical testing applicable to full communities | 1 - Unintentionally subjecting specific areas to an the possibility of a disruptive intervention (lockdowns, quarantines, isolations of entire areas without any process for identifying and isolating relevant individuals; mandatory testing of individuals in response to wastewater data) whereas excluding others from the same level of surveillance scrutiny and response | 2 - Intentionally subjecting specific areas to an the possibility of a disruptive intervention (lockdowns, quarantines, isolations of entire areas without any process for identifying and isolating relevant individuals; mandatory testing of individuals in response to wastewater data) whereas excluding others from the same level of surveillance scrutiny and response |
| 3 | **Trust:** Does WBS violate assumptions that are made about how personal info is treated?* | 0 - Wastewater treatment facility/water resources recovery facility influent testing from public infrastructure with large geographical service area of a target that does not identify individuals | 1 - Testing via maintenance holes at the sub-sewershed and neighborhood level and/or the surveillance target may uniquely monitor a protected class | 2 - Testing from individual buildings or private property and/or the surveillance target is designed to uniquely monitor a protected class (or individual) |
| 4 | **Invalidity:** Does WBS produce invalid results?* | 0 -Use of validated workflow/analysis and QA/QC; finalized results have been validated to correlate with clinical outcomes; routinely monitored chain of custody of samples and data; data is properly matched to the underlying engineering context of the collection system | 1 - Omissions from validation and QA/QC; positive preliminary results coincide with clinical outcomes, correlation requires further data; data acknowledges the influence of the collection system, but may not be fully matched in terms of combined, geography, or other parameters | 2 - Non-validated workflow with no QA/QC; no relationship with the clinical outcomes; no chain of custody; no attempt to contextualize in terms of the collection system |
| 5 | **Awareness:** Are individuals informed they are being monitored and why?* | 0 - Representative(s) of the monitoring campaign are in a cycle of continued community outreach and engagement during the sample collection and over the clearly defined reporting period providing contextualization of the scope and intent to minimize misrepresentation or misuse (data is covered under 12); those monitoring capture the questions from the community rather than the wastewater utility operators | 1 - Duration, scope, and intent is communicated and disseminated in a passive manner without contextualization or engagement or communicated to a single representative of the community; the wastewater utility operators field increased inquiries but has access to those collecting the data to point inquiries to | 2 - No direct communication of the duration, scope, and intent to the monitored community members; the burden of communication falls solely on the third-party wastewater utility operators rather than the data collectors |
| **ID** | **Category** | **0 - Minimal Review Required** | **1 - Review Suggested** | **2 - Review Strongly Suggested** |
| 6 | **Consent:** Have the primary monitored population been given equal and accessible means to provide consent, and have those that withheld been given means to be excluded from monitoring?* | 0 - Received formal consent from the community monitored (e.g., city level consent can be from elected public health officials, through democratic referendums; sewersheds - community board votes; individual properties - written consent of owners/inhabitants) and/or collecting non-directly identifiable data; when consent is withheld, monitoring of those withholding consent is not performed | 1 - Received proxy consent from the community monitored (e.g., formal independent review of the collection method provides approval from un-elected community stakeholder leaders, property owners but not inhabitants providing approval) while collecting non-directly identifiable data OR following a pooled-method approach with independent stakeholder review when collecting potentially directly identifiable data; when consent is withheld, those withholding consent cannot be identified/targeted within the performed monitoring | 2 - Direct target of identifiable data without independent review and/or without community consent in any form; those withholding consent remain monitored and identifiable and/or the monitored community has explicitly withdrawn consent, but is still being monitored |
| 7 | **Golden Rule:** Would/do those performing or authorizing WBS subject themselves to the monitoring **Silver Rule:** Would/do those performing or authorizing WBS perform surveillance that they would object to?* | 0 - Those monitoring & authorizing monitoring are monitored and consent to the performed surveillance | 1 – Those monitoring & authorizing monitoring are not monitored but would consent to the performed surveillance | 2 - Those monitoring & authorizing monitoring are not monitored and would not consent to the performed surveillance and/or those monitoring are monitored do not consent to the performed surveillance |
| 8 | **Minimization:** Is the minimal amount of observation collected to address the public health need?* | 0 - Only the data that is clearly communicated in the initial scope of the project description that matches the public health goal is extracted from the sample. Sample is promptly destroyed. | 1 - Only the data that is clearly communicated in the initial scope of the communicated project description that matches the public health goal is extracted from the sample. Sample is not promptly destroyed. | 2 - Samples are kept indefinitely with no articulated or approved scope for further data collection |
| 9 | **Public Decision-Making:** Was the decision to use WBS arrived at through some public discussion and decision-making process?* | 0 - Surveillance program was designed in a public manner (e.g., review by elected officials and public through town halls for initial implementation and continued operation) with a good-faith effort to reach both those receptive or resistant to the objectives of public health | 1 - Program does not receive formal public authorization but is broadly supported by the public (as informed by representative public surveys) | 2 - Program does not receive formal public authorization and is not supported by the public (as informed by representative public surveys) |

| **ID** | **Category** | **0 - Minimal Review Required** | **1 - Review Suggested** | **2 - Review Strongly Suggested** |
| --- | --- | --- | --- | --- |
| 10 | **Human Review:** Is there human review of machine-generated results?* | 0 - Action is taken based on contextualizing wastewater data, other public health data, and institutional-oversight of human review (e.g., alert sent after wastewater concentrations exceeds a certain threshold, cases exceeds a threshold, and a human reviews it following guidelines established and agreed upon in the scope of work); QA/QC of any automated processes clearly identified and repeated throughout the campaign; contextualized in terms of the underlying engineering context of the collection system | 1 - Action is taken based on wastewater data alone and human review without institutional-oversight (e.g., alerts sent when wastewater concentrations exceeds a threshold & a human reviews the alert without prior agreed upon guidelines); QA/QC of automated processed only mentioned; contextualizing to the underlying engineered structure of the collection system has select gaps | 2 - Action is taken automatically based on wastewater data (e.g., automatic alerts when wastewater concentrations exceeds a threshold); no QA/QC of automated processes; no contextualization to the underlying engineered structure of the collection system |
| 11 | **Right of Inspection:** Are people aware of the findings of WBS and how they were created?* | 0 - Representative(s) of the monitoring campaign are in a cycle of continued community outreach and engagement during the sample collection and reporting period providing contextualization of the collected data to minimize misrepresentation or misuse (e.g., updating an annotated and agreed-upon internet-accessible dashboard; timely and routine public town-halls or open seminars; direct mailing to surveyed individuals) | 1 - The collected data is communicated and disseminated in a passive manner without contextualization or engagement or communicated to a single representative of the community | 2 - No direct communication of the collected data to the monitored community members |
| 12 | **Right to Challenge:** Are there procedures for challenging the results of WBS?* | 0 - Grievance procedure is established with an ability to appeal to opt-out; those monitoring are or have designated the point person to handle these grievances | 1 - Grievance procedure is established only to collect community feedback; those monitoring are available to handle these grievances | 2 - No grievance procedure or contact information provided; wastewater utility operators shoulder the responsibility solely for handling these grievances |
| 13 | **Redress and Sanctions:** If the individual has been treated unfairly and procedures violated, are there appropriate means of redress? Are there means for discovering violations and penalties to encourage responsible surveillant behavior?* | 0 - Clearly defined oversight of the surveillance campaign with established metrics for determining inappropriate or technically unsound behavior with associated penalties (e.g., contract cancellation, community compensation, paid medical data security services, etc.) | 1 - Oversight with unstructured penalties available | 2 - No oversight and/or no penalties available |
| 14 | **Equality-inequality:** Is the means to conduct WBS widely available?* | 0 -Wastewater program implemented with attention toward equal representation of all relevant constituents | 1 - No explicit consideration of equality or representativeness during program implementation | 2 - Program implemented in such a way as to be deliberately inequitable; designed inequality, for example excluding high-GDP per capita areas seeking exemption or including only these same areas |

| **ID** | **Category** | **0 - Minimal Review Required** | **1 - Review Suggested** | **2 - Review Strongly Suggested** |
| --- | --- | --- | --- | --- |
| 15 | **Equality-inequality:** Is WBS broadly applied to all or only those able to resist?* | 0 - Entire community is monitored (e.g., treatment plant; jail sampling that monitors the effluent of the whole jail including staff and inmates) | 1 - Representative coverage is achieved (e.g., manhole sampling, but ensuring that demographics of surveilled communities are representative of the entire city; jail sampling that has sites for staff and inmates separately) | 2 - Only protected-class communities are monitored (e.g., manhole sampling that surveils only low-GDP per capita areas; jail sampling that only surveils inmates) |
| 16 | **Equality-Inequality:** Is resistance to WBS equally available?* | 0 - Community-wide surveillance program inclusive of all areas serviced by the wastewater utility and clearly articulated methods of public comment and oversight | 1 - Lack of clearly articulated inclusion/exclusion criteria and procedures for public disclosure, comment, and feedback | 2 - Political power leveraged to exclude or include certain areas with or without their permission |
| 17 | **Negative Effects of Surveillance on Third Parties:** Are there negative effects on those beyond the subject and, if so, can they be adequately mediated?* | 0 - After performing a risk analysis, no negative effects on those outside the monitoring campaign are identified | 1 - After performing a risk analysis, potential indirect negative effects on third parties are identified (e.g., competition of resources during supply chain limitations) | 2 - After performing a risk analysis, potential direct negative effects on third parties (e.g., monitoring comingled wastewater from those outside the intended campaign, damaging the sewer system or otherwise preventing access, shifting risk of exposure to sample collectors) |
| 18 | **Beneficiary:** Does application of WBS serve broad community goals, the goals of the object of surveillance, or the personal goals of the data collector?* | 0 - Program data used to inform community-level decision making relevant to public health | 1 - Data are used to do nothing OR public program data used for private means (e.g., management of work force or personnel) | 2- Public program data used for private profit and/or inaccessible or unaffordable for public entities |
| 19 | **Community Values:** Are the values and concerns of the communities taken into account in planning, implementing, and using data from surveillance?† | 0 - Representative of the monitoring campaign are in a cycle of continued community outreach and engagement during the planning and implementing period to address the concerns and support the values of the community | 1 - Representative of the monitoring campaign are engaged during the planning period only to address the concerns and support the values of the community | 2 - No direct involvement of the monitored community members |
| 20 | **Proportionality:** Are the resources committed proportional to the public health need?* | 0 - Cost and resource efficient program with strong oversight from public officials; clear reporting on financials; clear use case for resulting data | 1 - Cost and resource efficient program with strong oversight from public officials and clear reporting on financials without a clear use case for resulting data; large expense of programs with substantial need of the resulting data stream | 2 - Large expense of programs with little to no use of the resulting data stream; poor definition of goals, objectives, and how data will be used; for internal private research and development only |
| 21 | **Alternate Means:** Are other, less burdensome means available?* | 0 - There are no other data sources for this surveillance purpose | 1 - There exist other, but incomplete or delayed, data sources (e.g., opioid overdoses, Covid-19 case data) | 2 - There exists other complete data for this same purpose (e.g., 100% individual-testing compliance for a virus in a pandemic) |
| 22 | **Consequence of Inaction:** What are the consequences of taking no surveillance action?* | 0 - Mortality, morbidity, or other adverse effects is imposed on community by lack of surveillance | 1 - The surveillance data does not minimize adverse effects to the community | 2 - The community benefits by not being surveyed |
| **ID** | **Category** | **0 - Minimal Review Required** | **1 - Review Suggested** | **2 - Review Strongly Suggested** |
| 23 | **Protection:** Are adequate steps taken to minimize costs and risk?* | 0 - Full internal review performed and assessed by an external auditor; cost justification and alternatives analysis considered with input/review by public; competitive bidding for programs; data security program in place | 1 - Full internal review performed but not assessed by an external auditor; data security plan in place | 2 - No cost oversight or justification; price gouging; no competitive bidding; no data security |
| 24 | **Legitimacy:** Are surveillance data collected only for a legitimate public health purpose?† | 0 - Data is collected by public health agents for public health measures | 1 - Data is collected by non-public health agents for public health measures | 2 - Data is collected by non-public health agents (or public health agents acting outside of public health) for non-public health purposes |
| 25 | **Appropriate vs. Inappropriate Goals:** Are the goals of the data collection legitimate?* | 0 - Goals clearly articulated and subjected to review by agents of the public | 1 - Goals poorly or imprecisely defined (duration, targets, purpose, etc.) but oversight and review in place | 2 - No articulated goals or oversight |
| 26 | **Feasible and Sustainable:** Does the surveillance system have a clear purpose and a plan for data collection, analysis, use, and dissemination based on relevant public health priorities?† | 0 - The information collected from wastewater directly supports a clearly-defined public health outcome | 1 - The information collected from wastewater potentially supports a general public-health outcome | 2 - The information collected from wastewater does not support a public health outcome |
| 27 | **Means Matching the Goal:** Is there a clear link between the information collected and the goal sought?* | 0 - The information collected from wastewater directly supports a clearly-defined public health outcome | 1 - The information collected from wastewater potentially supports a general public-health outcome | 2 - The information collected from wastewater does not support a public health outcome |
| 28 | **Information Used for Original Purposes:** Is the data used for the reasons offered for its collection and for which consent may have been given, and do the data stay with the original collector?* | 0 - Data is used only for the primary purpose | 1 - Data is used for secondary (e.g., research) purposes, with consent provided for any public use | 2 - Data is used for secondary purposes without consent |
| 29 | **No secondary gains:** Does the data generated or campaign itself benefit a secondary party?* | 0 - Data used only for the purposes approved and all uses performed under the oversight of officials/agents of the public; monitoring campaign does not benefit an external group | 1 - Data used for decision making relevant to management of persons without their review or input (e.g., managing employee wellbeing); monitoring campaign may benefit neighboring unmonitored regions (e.g., alterations in perception, property values) | 2 - Data repackaged or repurposed for profit (e.g., targeted medication ads, insurance sales); monitoring one community directly benefits an unmonitored community (e.g., stigma lowering property values in one region, increasing in another) |

| **ID** | **Category** | **0 - Minimal Review Required** | **1 - Review Suggested** | **2 - Review Strongly Suggested** |
| --- | --- | --- | --- | --- |
| 30 | **Identification:** Are names or identifiable data collected?† | 0 - Identifiable data collected only after satisfying community review and World Health Organization guidelines | 1 - Identifiable data collected only after satisfying World Health Organization guidelines | 2 - Identifiable data collected without consultation |
| 31 | **Data Stewardship and Protection:** Is the data properly maintained to protect those monitored?* | 0 - Data managed per requirements of and for community monitored (codeveloped with the community and professional practice) | 1 - Data managed per requirements of for community monitored (set by professional practice alone) | 2 - Data management plan absent |
| 32 | **Internal Data Sharing:** Do all parties involved in surveillance share data in a timely fashion?† | 0 - Data is shared between stakeholders after passing an agreed upon and optimized QA/QC timeline | 1 - Data is shared between stakeholders but with significant delays | 2 - Data is not shared |
| 33 | **External Data Sharing:** Is the public health surveillance data shared with other public health agencies when addressing a public health need?† | 0 - Collected wastewater-supported public health data is shared freely with/between public health agencies when a public health need presents or persists | 1 - Collected wastewater-supported public health data only partially shared with/between public health agencies when a public health need presents or persists | 2 - No data is shared when a public health need presents or persists |
| 34 | **External Data Sharing for Non-Public Health:** Is personally identifiable surveillance data shared with agencies that are likely to use them to take action against individuals or for uses unrelated to public health?† | 0 - Identifiable data is collected and shared only with public health officials to support public health targeted goals OR no identifiable data is collected | 1 - Identifiable data is collected and shared with multiple agencies to support public health | 2 - Identifiable data is collected and shared with agencies without any public health aim |
| 35 | **Further Research:** With appropriate justification and safeguards, do public health agencies use or share surveillance data for research purposes?† | 0 - Supported research directly benefits the public health initiative | 1 - Supported research may tangentially benefit the public health initiative | 2 - No clear benefit to the public health initiative is reasonably anticipated from the performed research |
| 36 | **Symbolic Meaning of Method:** What does the use of a method communicate more generally?* | 0 - Excrement from your body will be screened for disease as part of a collaborative partnership between the community and public health officials with oversight and input from the public under the framework of contaminant management without means to directly identify individuals | 1 - Excrement from your body is screened for disease without your permission and/or without an ability to opt out except through extraordinary individual effort (e.g., maintaining a separate on-sight sanitation facility such as an portable toilet) without means of directly identifying individuals | 2 - Excrement from your body will be screened for disease without your permission and/or without an ability to opt out except through extraordinary individual effort (porta potty) with means of directly identifying individuals |

| **ID** | **Category** | **0 - Minimal Review Required** | **1 - Review Suggested** | **2 - Review Strongly Suggested** |
| --- | --- | --- | --- | --- |
| 37 | **Creation of Unwanted Precedents:** Is it likely to create precedents that will lead to its application in undesirable ways?* | 0 - Analysis for individual identification prohibited; explorations outside of agreed-upon community scope is explicitly prohibited | 1 - No positional statement regarding individual identification; No discussion of future research is discussed | 2 - Explicitly for identification of individuals or otherwise unethical applications |
